# Computational Identification of Amino-Acid Mutations that Further Improve the Activity of a Chalcone–Flavonone Isomerase from *Glycine max*

**DOI:** 10.3389/fpls.2017.00248

**Published:** 2017-02-24

**Authors:** Hui Yuan, Jiaqi Wu, Xiaoqiang Wang, Jiakuan Chen, Yang Zhong, Qiang Huang, Peng Nan

**Affiliations:** ^1^Ministry of Education Key Laboratory for Biodiversity Science and Ecological Engineering, School of Life Sciences, Fudan UniversityShanghai, China; ^2^Department of Biological Sciences, University of North Texas, DentonTX, USA; ^3^Institute of Biodiversity Science and Geobiology, Tibet UniversityLhasa, China; ^4^State Key Laboratory of Genetic Engineering, School of Life Sciences, Fudan UniversityShanghai, China

**Keywords:** enzyme engineering, positive selection, protein design, molecular modeling, chalcone–flavonone isomerase

## Abstract

Protein design for improving enzymatic activity remains a challenge in biochemistry, especially to identify target amino-acid sites for mutagenesis and to design beneficial mutations for those sites. Here, we employ a computational approach that combines multiple sequence alignment, positive selection detection, and molecular docking to identify and design beneficial amino-acid mutations that further improve the intramolecular-cyclization activity of a chalcone–flavonone isomerase from *Glycine max* (GmCHI). By this approach, two GmCHI mutants with higher activities were predicted and verified. The results demonstrate that this approach could determine the beneficial amino-acid mutations for improving the enzymatic activity, and may find more applications in engineering of enzymes.

## Introduction

**FIGURE 1:**
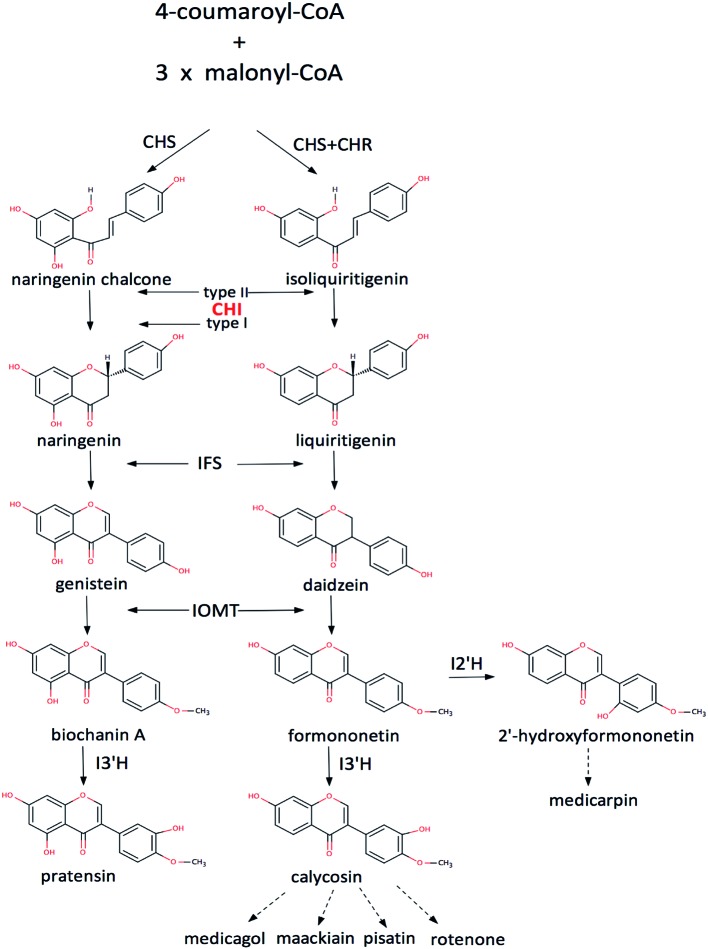
**The flavonoid pathway.** CHS, chalcone synthase; CHR, chalcone reductase; IFS, isoflavone synthase; IOMT, 2′-hydroxyisoflavanone 4′-*O*-methyltransferase; I2′H, isoflavone 2′-hydroxylase; I3′H, isoflavone 3′-hydroxylase (Liu and Dixon, 2001; Liu et al., 2002, 2003; Shimada et al., 2003).

Flavonoids are widespread secondary products in plants, especially in leguminous plants. They play important roles in plant physiology and ecology (Mol et al., 1998; Mahajan and Yadav, 2014), and are also important source of medicine and drug development (Dixon and Steele, 1999; Martens and Mithofer, 2005). Thus enzymes in the flavonoid biosynthetic pathways are of considerable value in biotechnological practices (Liu and Dixon, 2001; Liu et al., 2002, 2003). Of them, chalcone–flavonone isomerase (CHI) is an important enzyme in the biosynthetic pathway that catalyzes the intramolecular cyclization of a chalcone into a (2S)-flavonone (**Figure 1**, Supplemenatary Figure S1). According to their catalytic features, CHIs could be divided into two groups: type-I and type-II, respectively (Shimada et al., 2003). The type-II CHIs exist only in legumes and have broader substrate acceptability than the type-I enzymes (**Figure 1**), which are found in both non-legumes and legumes (Kimura et al., 2001; Shimada et al., 2003).

Because of their unique properties, there is a great demand of the flavonoids and their derivatives in biotechnology and medicine. However, the flavonoid production from natural plants could not fulfill such a demand. Therefore, it is desirable to develop biochemical and

biotechnological methods to synthesize novel derivatives and increase their production by improving the corresponding biosynthetic enzymes, such as the CHIs. Thus, the improvement of the CHI activity is not only important for understanding the molecular determinants of the enzymatic activity, but also significant for biotechnological applications. Currently, rational design and directed evolution are two major strategies (Barak et al., 2008; Damian-Almazo and Saab-Rincon, 2013). Based on structural information, rational design usually identifies target amino acids near the active site, and then carries out site-directed mutagenesis to obtain protein mutants with enhanced activity. As an alternative, directed evolution mimics the process of nature evolution or/and recombination to obtain better mutants of the enzymes (Barak et al., 2008). Although both methods have proven to be useful in protein design, they also have certain limitations (Grove et al., 2003; Chica et al., 2005; Chen et al., 2009). Therefore, it is necessary to use combined approaches in order to overcome such limitations (Funke et al., 2005; Damian-Almazo and Saab-Rincon, 2013).

In practice, it is very laborious and costly to experimentally test a large number of candidate mutants. Therefore, it is very important to accurately identify residue sites for the mutagenesis. To the end, both sequence-based and structure-based methods were used. A common sequence-based approach is the multiple sequence alignment (MSA) that is effective for identification of conserved sites, some of which are the target mutation sites toward better activity. For example, one could build a correlation between the sequence pattern observed in the MSA and enzymatic property (Damian-Almazo and Saab-Rincon, 2013), e.g., the analysis of subfamily specific positions (SSPs) (Suplatov et al., 2012). Another way is to examine the ancestral relationship among the homologous sequences by combing the MSA with phylogenetic information (Di Giulio, 2001, 2003). On the other hand, structure-based approaches usually focus on those sites that are in the vicinity of the catalytic residues or the substrates (Morley and Kazlauskas, 2005; Park et al., 2006; Paramesvaran et al., 2009). However, because these approaches usually identify too many mutagenesis sites, it is still hard to experimentally test all those sites. In addition, mutants with reduced activity are often generated using these approaches, and it is very difficult to design and generate mutants with enhanced activity, especially for those which wild-type enzymes exhibit high efficiency. Therefore, to accurately identify the mutagenesis sites and predict proper amino-acid types on those sites, it is necessary to develop new strategies.

**FIGURE 2:**
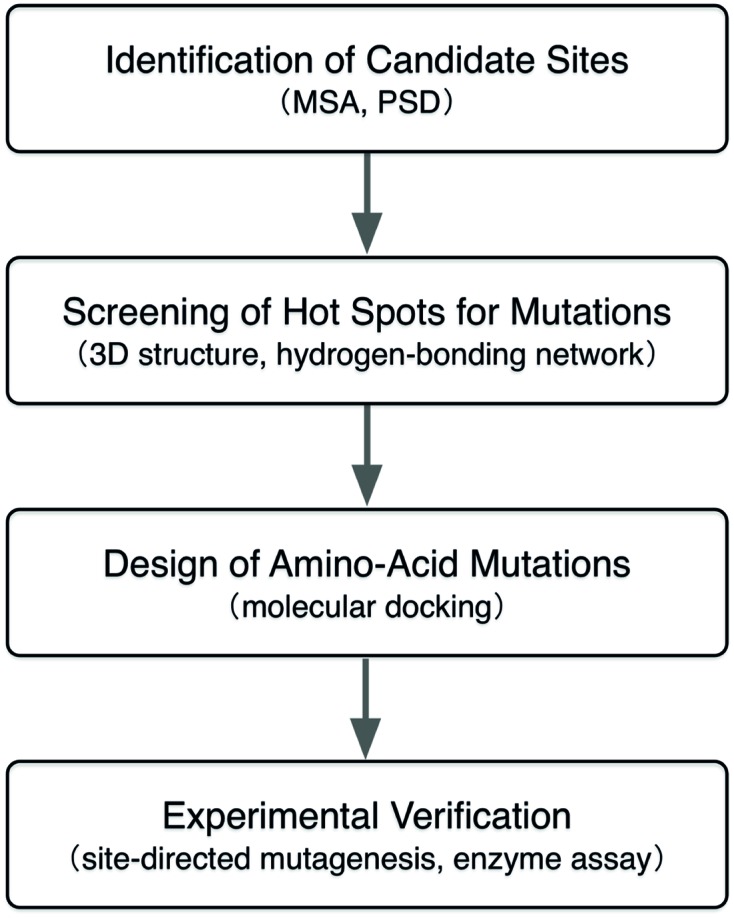
**Flowchart of the computational approach used in this study**.

In this study, we employed a computational approach that combines the MSA, evolutionally positive selection detection (PSD), and structure-based molecular docking to identify the beneficial amino-acid mutations for enhancing the intramolecualr-cyclization activity of a CHI from *Glycine max* (GmCHI, GI: 351723101), which is a type-II CHI and possesses high catalytic proficiency (k_cat_/K_m_ is about 5 × 10^6^ M^-1^ s^-1^). In the present study, as shown in **Figure 2**, candidate mutation sites in CHI enzyme were firstly identified using the MSA and PSD. Then, those candidate sites were investigated and further screened by analyzing the structural information and reaction mechanism. Next, the selected sites were further studied using molecular docking that determines the lowest-energy binding poses of the substrate in the active sites of the mutant enzymes. Finally, by *in vitro* assay using recombinant mutant enzymes, we identified beneficial amino-acid substitutions that improve the activity of GmCHI enzyme. Taken together, we demonstrate that our approach should be useful in designing of enzymes with improved enzyme activity.

## Materials and Methods

### Expression and Enzyme Assay of GmCHI Proteins

The ORF of GmCHI gene was cloned into an expression vector pET28a+ (Novagen^1^) and then expressed in *Escherichia coli* strain BL21 (DE3). The protein expression was induced by IPTG (1 mM) at 20°C, 180 rpm for 6–12 h. After expression, the cells were harvested and the protein was purified with Ni^2+^-NTA agarose (Bio-Rad^2^). The activities of the GmCHI proteins were measured according to the reaction kinetics of CHI enzymes. The substrate was incubated at 25°C, 90 s with total 500 μl reaction buffer (50 mM Tris, 500 mM NaCl, 1.0 mM DTT, pH 7.8) containing 5 ng of purified GmCHI protein. We performed the enzyme assays in a gradient concentration of 2–100 μM for isoliquiritigenin. After the reaction, the reaction mixtures including isoliquiritigenin and liquiritigenin were analyzed on an Agilent HP1100 HPLC with eclipse plus C-18 column. The eluents, consisting of 35% (v/v) acetonitrile and 0.1% (v/v) trifluoroacetic acid in water, were monitored at 276 and 372 nm (at constant flow rate of 1 ml per minute). The UV absorption values at 276 and 372 nm were used for quantifying liquiritigenin and isoliquiritigenin, respectively (He and Dixon, 2000; Liu and Dixon, 2001; Liu et al., 2002, 2003).

### Site-Directed Mutagenesis of GmCHI

The site-directed mutagenesis was performed using a mutagenesis kit from SBS Genetech^3^ and by following the manufacturer’s instructions. The primers used for the site-directed mutagenesis are listed in Supplementary Table S1. The mutants were confirmed by sequencing and then expressed in *Escherichia coli* according to the methods described above for the wild-type enzyme.

### Homology Modeling and Molecular Docking

We used the MODELLER program (Eswar et al., 2007) to build the all-atom structural models of the wild-type GmCHI protein and its mutants, with the crystal structure of MsCHI (PDB code: 1F7M) (Jez et al., 2000) as the template. Then, 2,000 independent, standard high-resolution refinement runs with the Rosetta program (Das and Baker, 2008) were carried out to generate refined atomic models with low free-enenrgies. For each GmCHI protein (the wild-type or the mutant), the 3D structure with the lowest free-energy from the 2,000 refined models was selected as the receptor structure for the subsequent molecular docking.

Hydrated ligand docking using the program AutoDock 4.2 (Forli and Olson, 2012) was conducted to predict the binding poses of the substrate in the active-sites of the wild-type GmCHI protein and its mutants. The hydration state of the substrate for the docking was determined and treated according to the reported method (Forli and Olson, 2012). All other docking parameters for the proteins and the substrate were set to the default values of AutoDock (Morris et al., 2009), with a size of the grid box around the active-site as 70 Å × 70 Å × 70 Å. The Lamarckian genetic algorithm was employed to search for the native-like binding pose, with a population number of 150, a maximum of 27,000 generations, and a maximum of 1,500,000 energy evaluations. To construct the binding energy landscape of the substrate in the active site of a GmCHI protein, 2,000 independent docking runs were performed, and thereby 2,000 binding poses were obtained for analysis and generating the RMSD-binding free energy plot.

## Results

### Identification of Amino-Acid Sites for Site-Directed Mutagenesis

#### Multiple Sequence Alignment (MSA)

**FIGURE 3:**
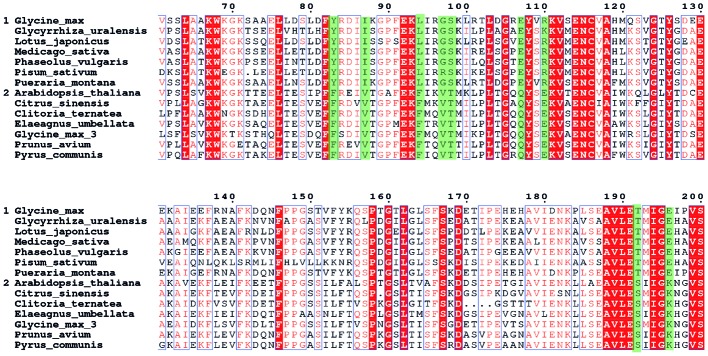
**The MSA of the CHI core regions.** The seven sequences in group 1 are type- II CHIs and another seven sequences in group 2 are type-I CHIs. The sequence positions are numbered according to GmCHI. The subfamily specific positions (SSPs) are marked in green.

Identification of the amino-acid sites for the site-directed mutagenesis is the first step toward the improvement of the GmCHI activity. To this end, we collected the sequences of 14 homologous CHIs (Supplementary Table S2), including seven type-II CHIs (group 1) and seven type-I CHIs (group 2), respectively. Then, we carried out the MSA for these CHIs, and thereby identified nine SSPs (**Figure 3**). On each of these sites, the wild-type amino acids are conserved within either the type-I or type-II group, but different between the type-I and type-II groups. Because the type-I and type-II CHIs are different in the catalysis (Kimura et al., 2001; Shimada et al., 2003), some of these sites might directly affect the reaction mechanisms and thus the enzymatic activity. Therefore, we considered them as the candidate sites for introducing beneficial amino-acid mutations.

#### Positive Selection Detection (PSD)

Besides the moderate conserved sites identified in the MSA, some particular unconserved sites under selection pressure (i.e., the positively selected sites) may also play a critical role in the evolution of protein function, because positive selection was considered to drive the fixation of advantageous mutations (Yang, 2006). Therefore, PSD was performed to further identify candidate sites for the mutagenesis. Firstly, we used RaxML 7.04 (Stamatakis, 2006; Stamatakis et al., 2008) under the GTR+Γ+I model to infer a phylogenetic tree of 15 CHI genes from 13 species, including eight type-I and seven type-II CHI genes. Then, based on this tree topology (Supplementary Figure S2) and branch-site model (Yang and Nielsen, 2002), the posterior probability of every site under positive selection was calculated with PAML 4.4 (Yang, 1997, 2007; Yang et al., 2005). Two sites (Val109 and Ile197) were identified as the positively selected sites, and then considered as the candidate mutation sites for improving the activity.

#### Screening by Structural and Reaction Information

By the above sequence-based methods, we have identified 11 potential sites for the mutagenesis. We further analyzed their spatial positions in the 3D structure obtained by homology modeling, and their effects on the hydrogen-bonding network in the active site required for the catalysis (Supplementary Figure S3). By manual inspection with the structural model (Supplementary Figures S4–S7), four target mutation sites were chosen, namely, Glu107, Ala110, Glu196 and Ile197, including conserved and unconserved sites (Supplementary Figure S8).

### Amino-Acid Mutation Design by Molecular Docking

**FIGURE 4:**
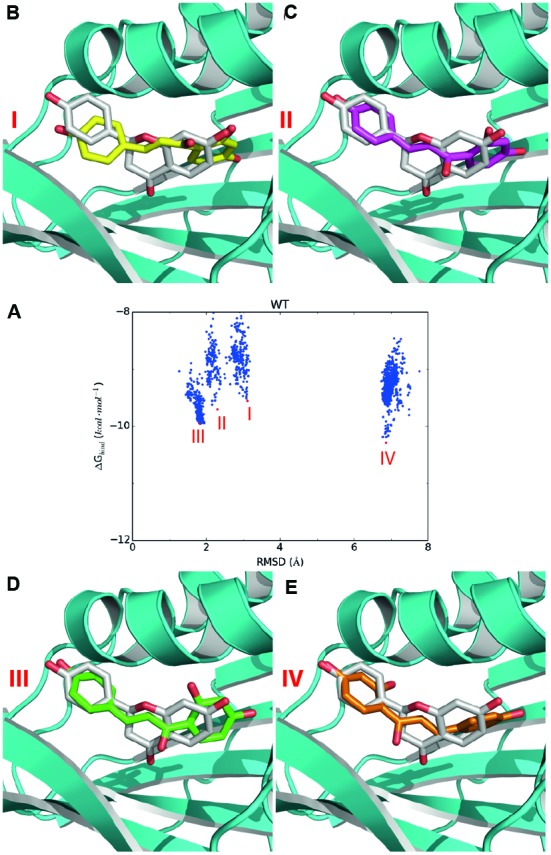
**Representative docking poses of the substrate in the active site of GmCHI.** **(A)** The RMSD-binding energy plot for GmCHI. According to the RMSDs with respect to the binding pose of the product, the docking poses could be divided to four groups: I, II, III, and IV. **(B–E)** The representative poses for groups I, II, III, and IV, respectively (binding pose of the product is shown in gray).

For the candidate mutation sites identified above, we further performed molecular docking studies to determine the types of amino acids for the substitution toward improving activity. For each site, we predicted the activities of the mutants with about five representative amino acids according to the physicochemical properties of their side chains, namely, a non-polar, an aromatic, a non-charged polar, an alkaline, and an acidic amino acid (for Glu196 and Ile197 on the loop, we predicted the acticity of the proline mutant). To predict the best amino-acid type at a given candidate site, we firstly constructed the 3D structure of a GmCHI mutant by the same homology modeling method for the wild-type enzyme. Then, we used the program AutoDock to dock the substrate (i.e., isoliquiritigenin) into the active site of the mutant by the protocols as described in Section “Materials and Methods.” Two thousand independent docking runs were conducted for each amino-acid mutation. Eventually, the RMSD values of the 2000 docking poses with respect to the product (i.e., liquiritigenin) (**Figure 4A**) were calculated and analyzed to identify the most likely amino-acid mutation.

To select the amino-acid type at the candidate sites, we firstly analyzed the RMSD-energy plot of the wild-type GmCHI obtained by molecular docking. As shown in **Figure 4A**, we found that the lowest-energy docking poses in the active site could be clustered into four main groups: groups I, II, III, and IV, respectively. For a given group, we selected the docking pose with the lowest binding energy in the group as the representative pose of the group. As indicated by the RMSD values, the representative binding poses of groups I, II, and III are very similar to that of the product revealed by the crystal structure (**Figures 4B–D**), whereas that of group IV possess an almost opposite orientation with a RMSD > 6.0 Å (**Figure 4E**). Moreover, the binding energies of the representative poses in groups II, III, and IV are lower than that of group I. Therefore, considering the binding to the active site is the initial step of the reaction, the most likely binding conformation of the substrate in the initial phase of the reaction is the representative pose of group I. Because of their similarity in the binding conformation of the product, the representative poses of groups II and III could be considered as intermediate conformations from the substrate to the product. Considering the order of the binding energy in three groups: group I > group II > group III, we may regard them as a transition states from the reactant to the product (**Figures 4B–D**). Thus, we hypothesized that, if an amino-acid mutation could further lower the binding energy of the representative pose of group III, the enzymatic activity of the mutant may be improved with respect that of the wild-type GmCHI.

**FIGURE 5:**
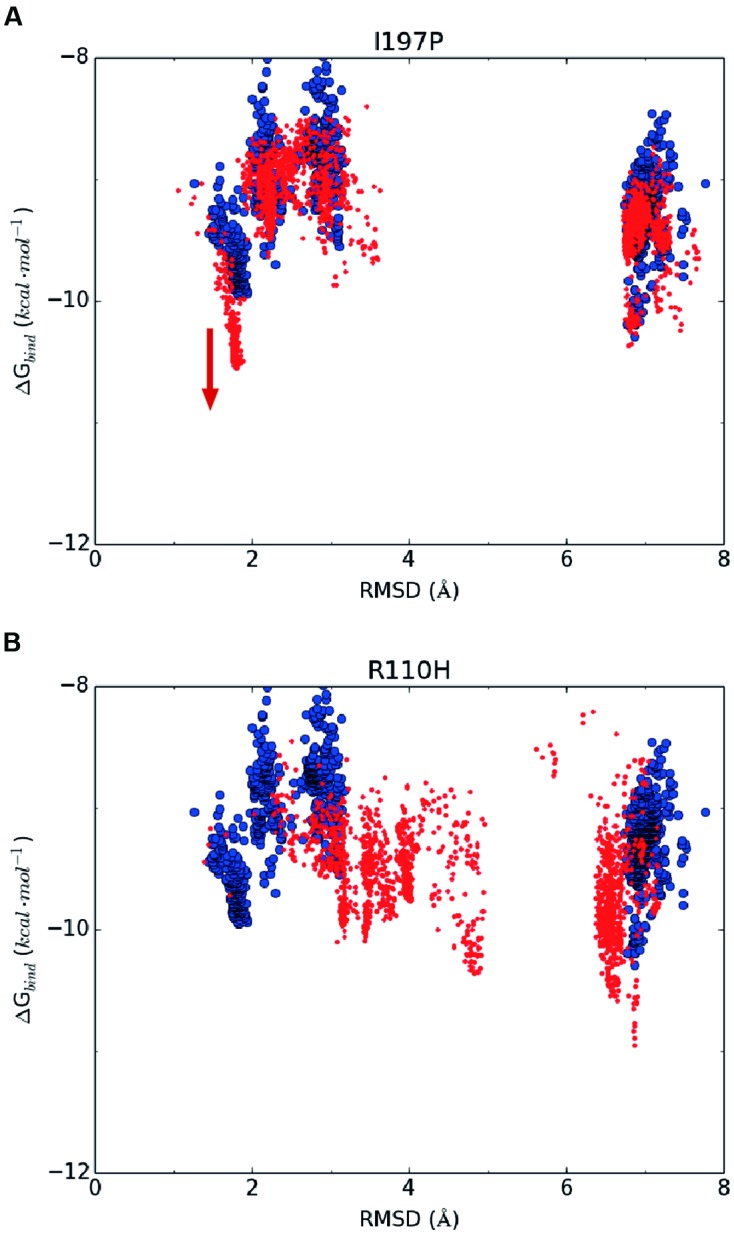
**The RMSD-binding energy plots of 2,000 docking poses of the wild-type enzyme (in blue dots) and the mutants (in red dots).** **(A)** I197P; **(B)** R110H.

Based on the above hypothesis, we generated the RMSD-binding free energy plots for the wild-type enzyme and all possible GmCHI mutants. Interestingly, we found that the binding energy of the I197P mutant with the representative pose of group III is significantly lower than that of the wild-type enzyme (**Figure 5A**), suggesting that this mutant might have higher activity than the wild-type enzyme. On the contrary, the corresponding energy of the R110H mutant is higher than that of the wild-type (**Figure 5B**), suggesting a decrease in the enzymatic activity with respect to the wild-type. Similarly, three mutants (E107D, R110A, and I197P) were also predicted to have higher activities than the wild-type (Supplementary Figures S9–S11).

### Molecular Cloning, Mutagenesis, and Enzyme Assay

According to the computational predictions, we carried out experiments to verify the activities of three beneficial GmCHI mutants (E107D, R110A, and I197P), as shown in Supplementary Figure S12. To the end, the wild-type GmCHI was cloned, and the site-directed mutagenesis was conducted on the target mutation sites. To examine the accuracy of the docking results, we also tested other mutants that represent various amino-acid types. We expressed the wild-type and mutant enzymes in *E. coli* expression system with BL21 (DE3) cells and purified them for the activity measurement (see Materials and Methods). The activities of all tested mutants are listed in **Table 1** (for more details see Supplementary Figures S13–S15). Compared with the wild-type enzyme, two mutants (R110A and I197P) do possess relatively higher activities, other mutants do not. This is consistent with the computational predictions (**Figure 5**, Supplementary Figures S9–S11). Significantly, the increase in the activity by the I197P mutation is about 53.3%, in good agreement with the prediction (**Figure 5A**). Thus, by using the computational approach in **Figure 2**, we identified two GmCHI mutants with higher activities.

**Table 1:** Activities of the GmCHI mutants with respect to that of the wild-type.

Enzyme	k_cat_/K_m_ (10^6^ M^-1^ s^-1^)	Change in activity (%)	*p*-value^∗^
Wild-type	4.963 ± 0.306	0.0	–
E107D	4.773 ± 0.336	-3.8	0.686
E107Q	3.715 ± 0.221	-25.1	8.82 × 10^-4^
R110A	7.0477 ± 0.412	42.0	3.24 × 10^-3^
R110E	4.142 ± 0.316	-16.6	0.046
R110H	3.345 ± 0.173	-32.6	2.98 × 10^-6^
I197P	7.608 ± 0.574	53.3	5.90 × 10^-4^


^∗^Calculated by covariance analysis using R software (http://www.R-project.org).

## Discussion

In this study, we have used a computational approach that integrates sequence-based analysis (MSA, PSD) and structure-based docking to identify the amino-acid hot spots for the site-directed mutagenesis, and then to predict the beneficial amino-acid mutations at those hot spots. The results demonstrate that the used approach could identify the beneficial amino-acid mutations that further improve the intramolecular-cyclization activity of GmCHI. Usually, when the catalytic proficiency of an enzyme (k_cat_/K_m_) reaches about 10^7^ M^-1^ s^-1^, diffusion rate becomes the main limition factor in the catalysis (Nelson and Cox, 2000). Then, it becomes difficult to further improve the catalytic proficiency. As mentioned, though the wild-type GmCHI has evolved to possess high catalytic proficiency (k_cat_/K_m_ = 5 × 10^6^ M^-1^ s^-1^.), the two GmCHI mutants predicted by our approach could further display a 50% increase in catalytic proficiency. Earlier researchers have employed bioinformatics methods for identifying potential target sites to be used for introducing mutations. These methods were very successful in the identification of those sites that stabilize enzymes (Damborsky and Brezovsky, 2014), e.g., thermostability (Sullivan et al., 2011; Anbar et al., 2012; Blum et al., 2012). However, they led to limited sucess in the identification of activity-related sites, because it is difficult to find out the functionally related sites based on sequence information alone.

Typically, to identify such sites, bioinformatics approaches are used for the analysis of moderately conserved sites, such as the SSPs (Suplatov et al., 2012). In our study, nine SSPs were identified in the MSA analysis, but only one of them was found to improve the enzymatic activity. Due to the natural selection, usually the wild-type amino acid at the conserved sites possesses the best activity, for example, mutations at site Glu107 do not improve the activity. Therefore, it remains very interesting to develop new methods that could identify functionally related sites from the unconserved sites.

The positively selected sites (Yang, 2006) are probably the most interesting unconserved sites, because they drive the functional divergence of many enzyme families during the evolution (Barkman et al., 2007; Lan et al., 2009, 2013; Huang et al., 2012), and could affect the activity or specificity of an enzyme (Kapralov and Filatov, 2007; Hao et al., 2010; Huang et al., 2012; Lan et al., 2013). Thus, PSD offers a way to predict the functionally related sites. In this study, two positively selected sites were detected, and the mutation at one of them was found to improve the enzymatic activity. Indeed, PSD is also able to identify functionally related sites that are not restricted in the vicinity of the active site, but distant away from the active site (Gillespie, 1991; Yang, 2006), e.g., Ile197.

On the other hand, to predict beneficial amino-acid mutations at the hot spots for the mutagenesis, here we used molecular docking as a fast method to search for such mutations, instead of using the computation intensive QM/MM methods. As demonstrated by the results, the predictions for almost all the six mutants are consistent with the experimental results (**Table 1**; Supplementary Materials see Enzyme assay). No doubt, this design strategy could also be used to improve other enzymes whose complex structures with the products have already been solved.

## Conclusion

To improve the GmCHI activity, we used a computational approach that combines sequence-based analysis with structure-based docking to identify the hot spots for amino-acid mutations and deign beneficial mutations at those sites. We successfully discovered two GmCHI mutants display higher activities than that of the wild-type enzyme. Because of its simplicity and low computational cost, this approach may find more applications in the design and engineering of enzymes.

## Author Contributions

PN and QH conceived and designed the study, and revised the manuscript; HY prepared samples, analyzed data, drafted the manuscript, and performed experiment verifications. JW performed the PSD; XW and QH directed the analysis of CHI structural models and molecular docking; JC and YZ provided constructive advices on the study. All authors read and approved the final manuscript.

## Conflict of Interest Statement

The authors declare that the research was conducted in the absence of any commercial or financial relationships that could be construed as a potential conflict of interest.

## Footnotes

1 http://www.novagen.com

2 http://www.bio-rad.com

3 http://www.sbsgene.com
