# Supplementary material for: Computational Identification of Amino-Acid Mutations that Further Improve the Activity of a Chalcone–Flavonone Isomerase from Glycine max
Source: Front Plant Sci. 2017 Feb 24;8:248. doi: 10.3389/fpls.2017.00248 (PMC5323383; doi:10.3389/fpls.2017.00248)
Supplement: Supplementary file 1 [file Presentation_1.PDF]

Supplementary Material

for

Computational identification of amino-acid mutations that further improve the activity of a chalcone-flavonone isomerase from *Glycine max*

Hui Yuan[1], Jiaqi Wu[1], Xiaoqiang Wang[2], Jiakuan Chen[1], Yang Zhong[1, 3], Qiang Huang[4, 5*] and Peng Nan[1*]

[1] *Ministry of Education Key Laboratory for Biodiversity Science and Ecological Engineering, School of Life Sciences, Fudan University, Shanghai 200438, China.*

[2] *Department of Biological Sciences, University of North Texas, Denton, TX 76203, USA*

[3] *Institute of Biodiversity Science and Geobiology, Tibet University, Lhasa 850000, China*

[4] *State Key Laboratory of Genetic Engineering, School of Life Sciences, Fudan University, Shanghai 200438, China.*

[5] *Shanghai Collaborative Innovation Centre for Biomanufacturing Technology, Shanghai 200237, China.*

*Correspondence and requests for materials should be addressed to P.N. (nanpeng@fudan.edu.cn) or Q.H. (huangqiang@fudan.edu.cn)

Contents **Page**

S1. Materials

S1.1. Site-directed mutagenesis of GmCHI gene S2

S2. Results

S2.1. The flavonoid pathway and reaction mechanism of CHI S2

S2.2. Multiple sequence alignment and positive selection detection S3

S2.3. Homology modeling of GmCHI protein structures S4

S2.4. Screening of amino acid sites for site-directed mutagenesis S6

S2.5. Molecular docking S9

S2.6. Enzyme assay S12

S1. Materials

1.1. Site-directed mutagenesis of GmCHI gene

Table S1. Primers of target sites for the site-directed mutagenesis

Primer	Sequence (5' to 3')
E107D	CATTGGATGGTCGT**GAT**TACGTAAGGAAGGTAT
E107Q	CATTGGATGGTCGT**CAA**TACGTAAGGAAGGTAT
R110A	ATGGTCGTGAATACGTA**GCG**AAGGTATCAGAGAACT
R110E	ATGGTCGTGAATACGTA**GAG**AAGGTATCAGAGAACT
R110H	ATGGTCGTGAATACGTA**CAC**AAGGTATCAGAGAACT
I197P	GACTATGATCGGAGAG**CCT**CCTGTTTCCCCTGC

S2. Results

S2.1. The flavonoid pathway and reaction mechanism of CHI

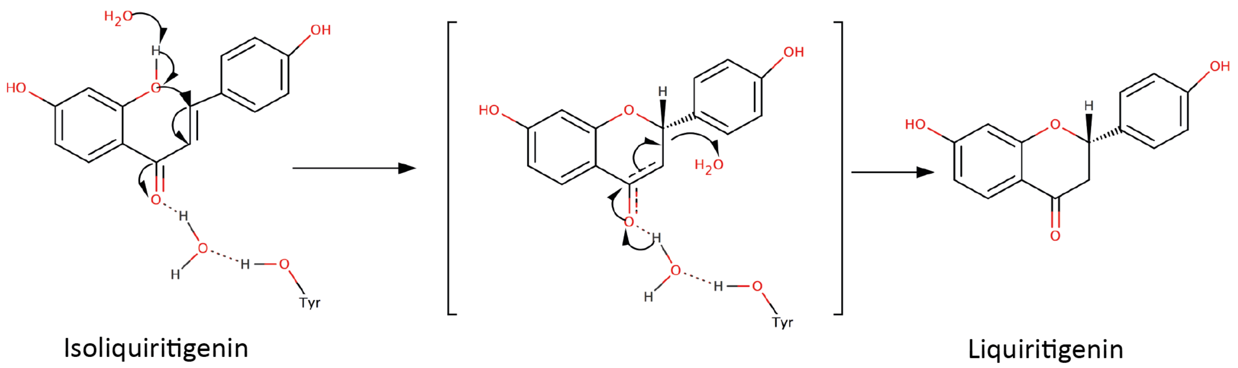

Figure S1 The reaction mechanism of CHI from *Medicage sativa* (MsCHI) (Jez et al., 2000; Jez and Noel, 2002). A key threonine (Thr48) triggers deprotonation of the substrate 2'-hydroxyl group to form oxyanion; then, the intramolecular nucleophilic attack of the oxyanion on the α, β-unsaturated double bond of the substrate results in the formation of a flav-3-en-4-ol intermediate, which is also stabilized by an tyrosine (Tyr106) through a water; finally, the intermediate tautomerizes into the product.

S2.2. Multiple sequence alignment and positive selection detection

Table S2. CHI sequences for the multiple sequence alignment.

GI	Species	*CHI's Type*
351723101	*Glycine max*	Type II (*Leguminosae)*
27530707	*Lotus japonicus*	Type II (*Leguminosae)*
166398	*Medicago sativa*	Type II (*Leguminosae)*
432404	*Pisum sativum*	Type II (*Leguminosae)*
20982	*Phaseolus vulgaris*	Type II (*Leguminosae)*
319759282	*Pueraria montana*	Type II (*Leguminosae)*
122725493	*Glycyrrhiza uralensis*	Type II (*Leguminosae)*
75151772	*Lotus japonicus*	Type I (*Leguminosae)*
351723469	*Glycine max*	Type I (*Leguminosae)*
133874176	*Clitoria ternatea*	Type I (*Leguminosae)*
3126969	*Elaeagnus umbellata*	Type I (non-*Leguminosae)*
71979902	*Fragaria x ananassa*	Type I (non-*Leguminosae)*
347326497	*Prunus avium*	Type I (non-*Leguminosae)*
227437128	*Malus hybrid cultivar*	Type I (non-*Leguminosae)*
500181573	*Pyrus communis*	Type I (non-*Leguminosae)*

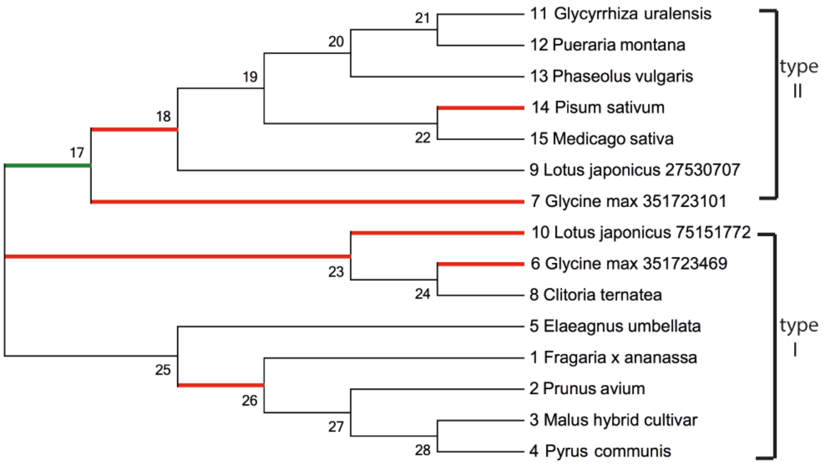

Figure S2. Phylogenetic tree of CHIs. There are three branches, type-I clade of leguminous plants and non- leguminous plants, type-II clade of leguminous plants (The nodes are marked by serial numbers).

Positive selection detection. The positively selected sites have a significant higher nonsynonymous substitution rate (d_N) than synonymous substitution rate (d_S). Usually, the nonsynonymous/synonymous rate ratio, $\omega = d_N/d_S$, is used to measure selective pressure. A significantly high nonsynonymous/synonymous rate ratio ($\omega > 1$) implies that the nonsynonymous mutation is favored by Darwinian selection (Yang, 2006). Therefore, the corresponding amino acid sites might play critical role in the evolution of protein function. Based on the tree topology (Fig. S2), positively selected branches and sites were detected by using branch-site model (Yang and Nielsen, 2002) and Codeml program of PAML (Version 4.4; Yang, 2007). Then likelihood ratio test (comparing with the null model) suggested the presence of sites under positive selection on the branch 17-7. To determine where these sites are located, the posterior probability that each site is under positive selection was calculated by using Bayes empirical Bayes approach (Yang et al., 2005). Two amino-acid sites of GmCHI were identified as positive selection sites: Val109 and Ile197 (corresponding probabilities are about 0.95).

S2.3. Homology modeling of GmCHI protein structures

Since GmCHI is a type-II CHI, structural information and reaction mechanism of homologous type-II CHIs are enlightening for understanding GmCHI. Therefore, we constructed the 3D structure of GmCHI using MODELLER (Eswar et al., 2007) with the crystal structure of MsCHI. (PDB code: 1F7M).

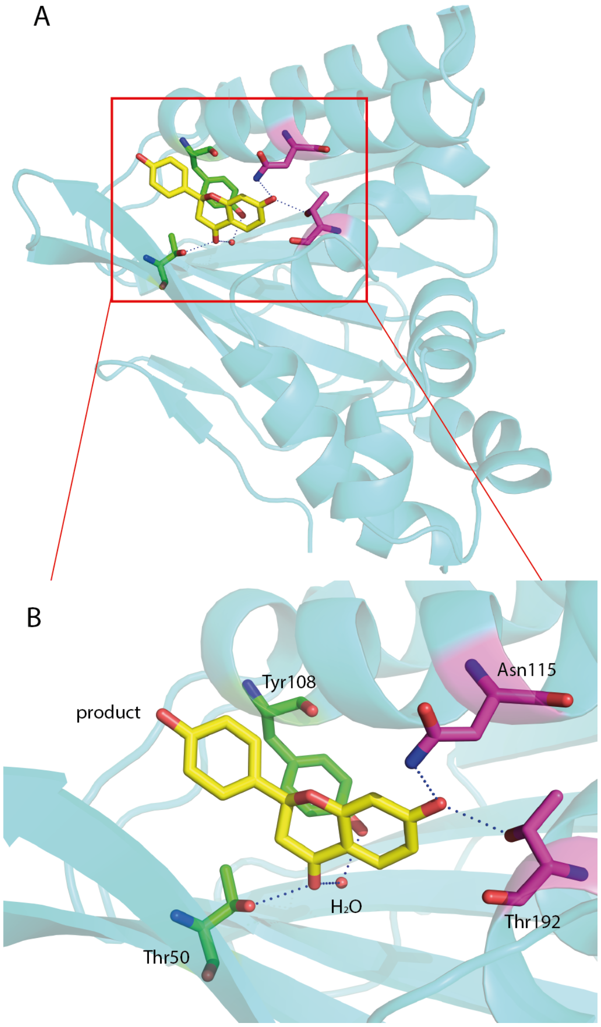

Figure S3. Three-dimensional structure of GmCHI by homology modeling. (A) Overall 3D structure of the GmCHI protein in complex with its product. (B) The active site of the GmCHI enzyme. Four key amino acids and a water molecule participate in the catalysis. These four amino acids and the hydrogen-bonding network are the same as those of MsCHI, except for a shift of 2 amino acids in sequence numbering: a threonine (Thr48 for MsCHI, Thr50 for GmCHI) that polarizes the ketone of the substrate, a tyrosine (Tyr106 for MsCHI, Tyr108 for GmCHI) that stabilizes the water molecule critical for the catalysis, an asparagine and a threonine (Asn113 and Thr190 for MsCHI, Asn115 and Thr192) that provide additional interactions for stabilizing the transition state (Jez et al., 2002).

S2.4. Screening of amino acid sites for site-directed mutagenesis

Several sites are not considered as the target mutation sites, because they are far away from the active site (Fig. S5), or their side-chains point away from the active site (Fig. S6), or they are very likely to break the hydrogen bond network (Fig. S7).

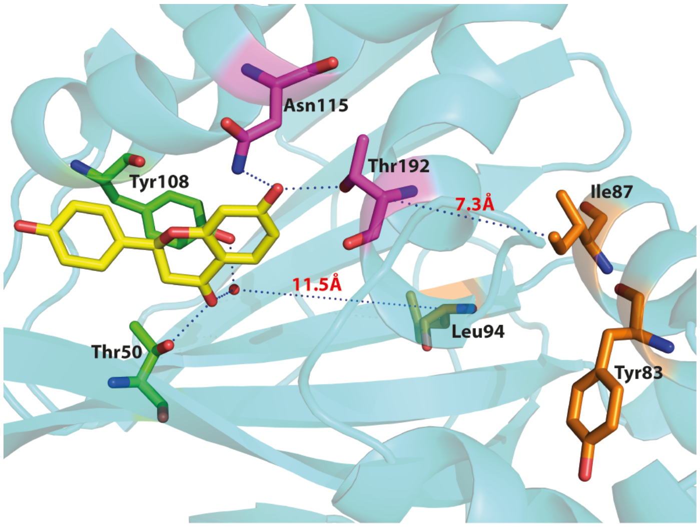

Figure S4. The distances between four candidate amino acid sites and active sites (Thr50, Glu108, Asn115, and Thr192).

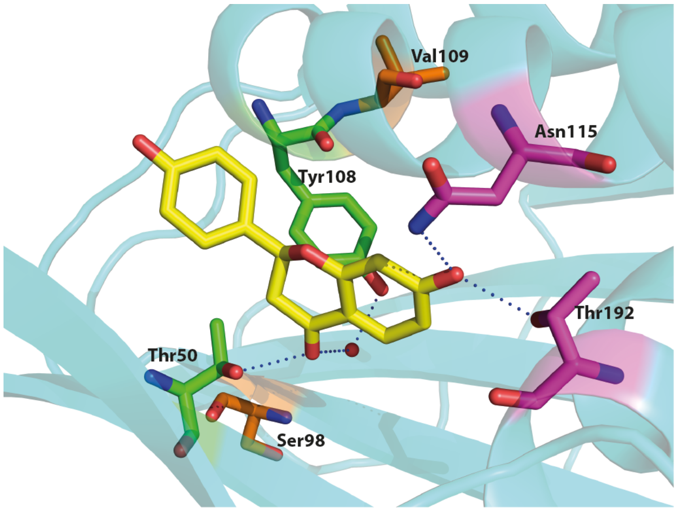

Figure S5. The orientations of the three candidate amino acid sites: Ser98 and Val109.

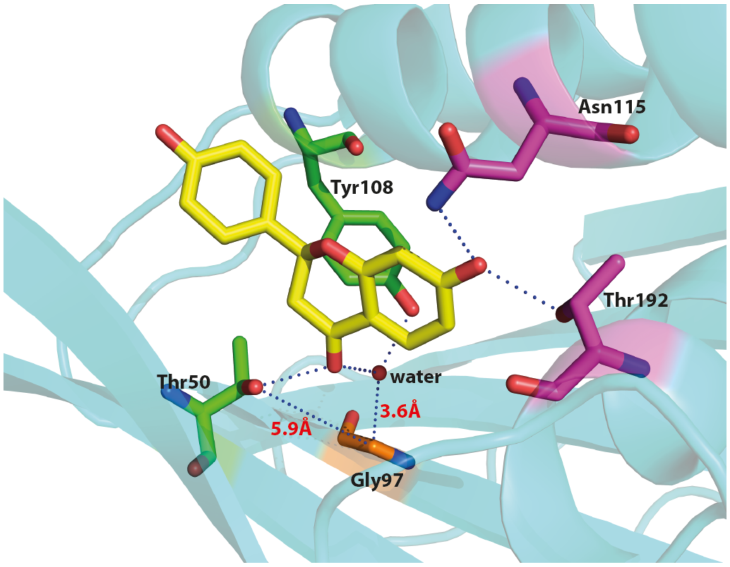

Figure S6. The candidate Gly97 might affect the hydrogen-bonding network, because Gly97 is very close to the catalytic water molecule.

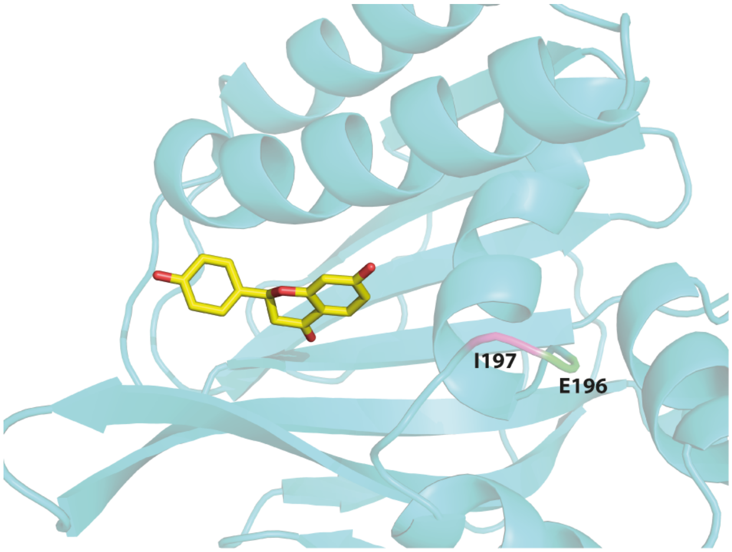

Figure S7. Two amino acid sites (Glu196 and Ile1197) are located at a loop that links an α-helix close to the active site. So, mutation of these two amino acids, especially proline, might alter the enzymatic activity by changing the loop conformation.

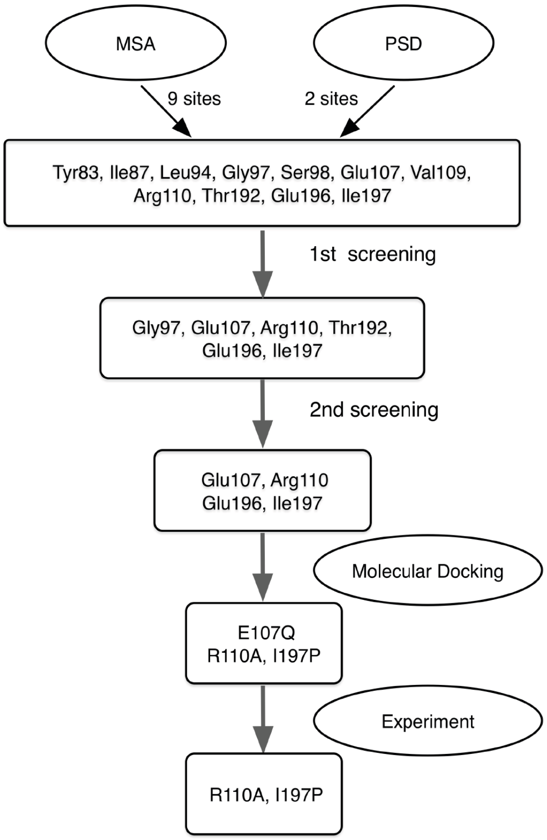

Figure S8. Workflow for the design of GmCHI mutants

S2.5. Molecular docking

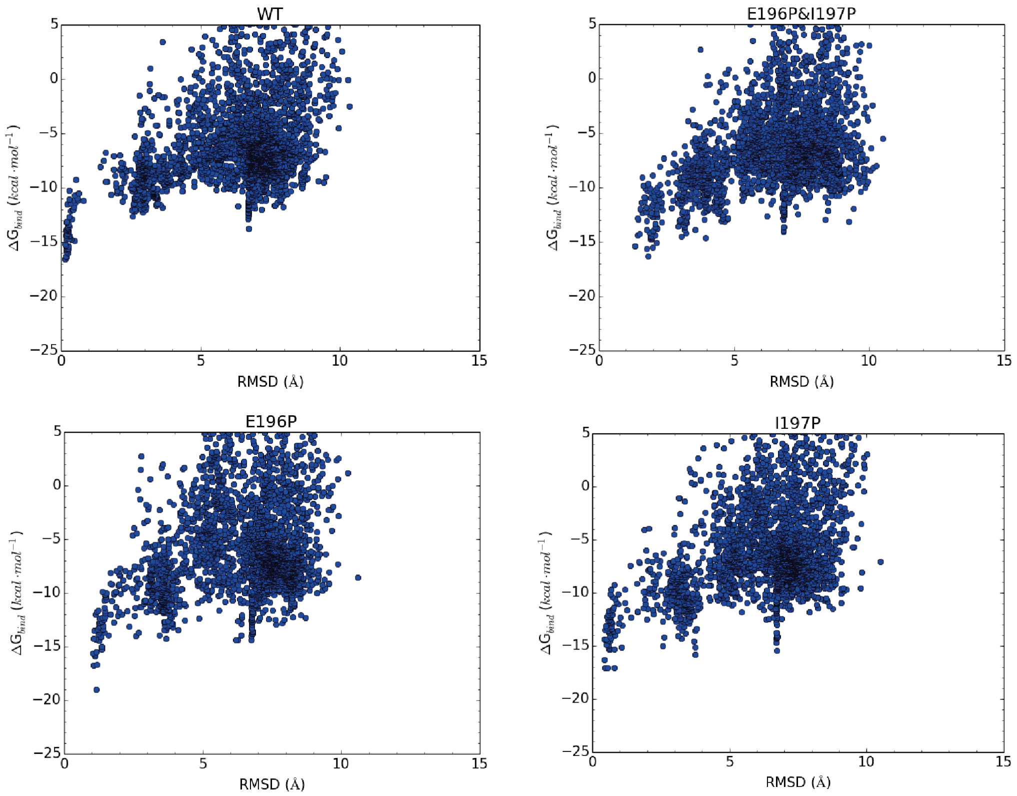

Figure S9. The RMSD-binding free energy plots of mutants at sites 196 and 197 with respect to the lowest-energy pose from 5,000 independent runs.

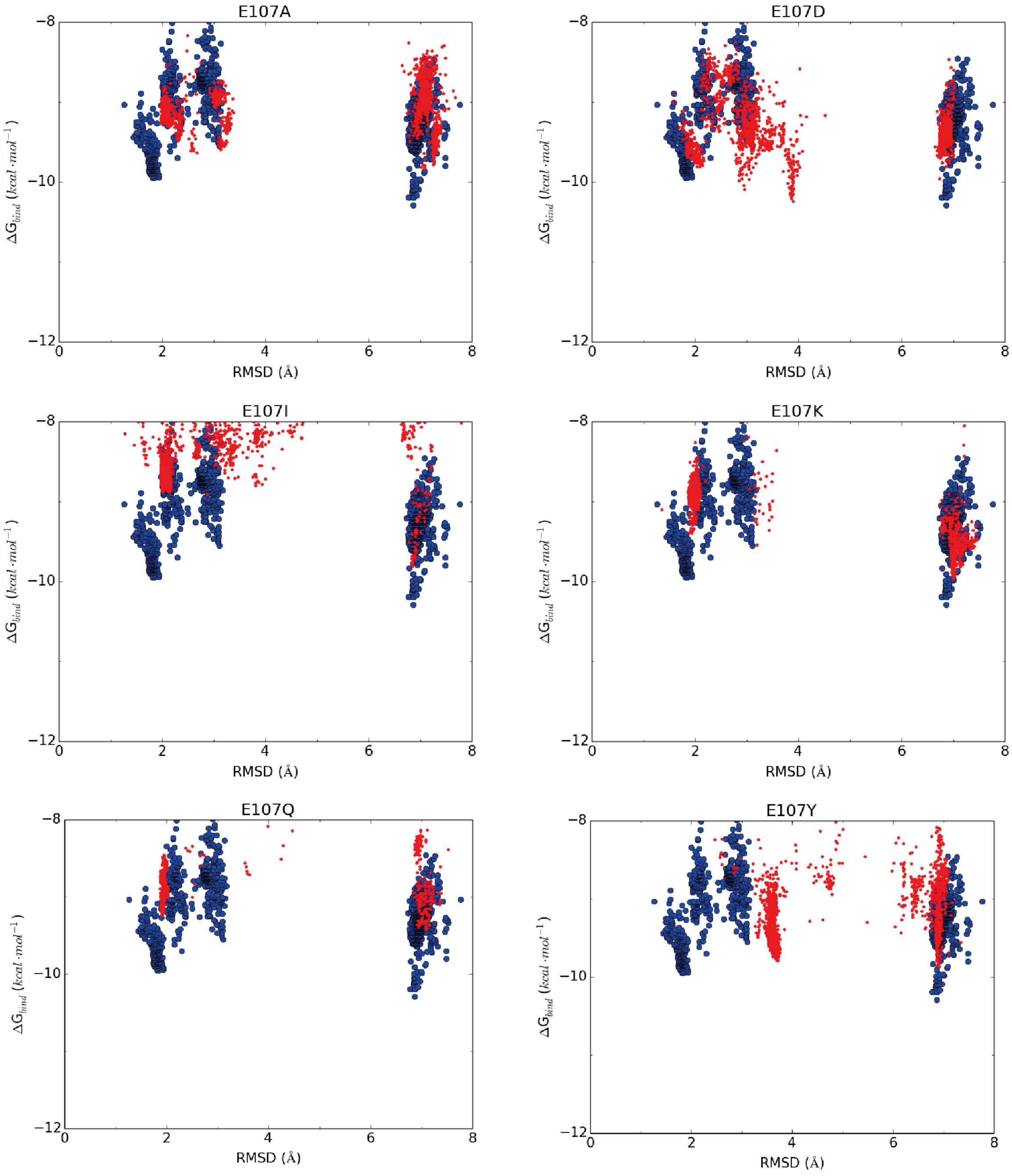

Figure S10. The RMSD-binding free energy plots of mutants (red dots) at site 107 with respect to that of the wild-type (blue dots).

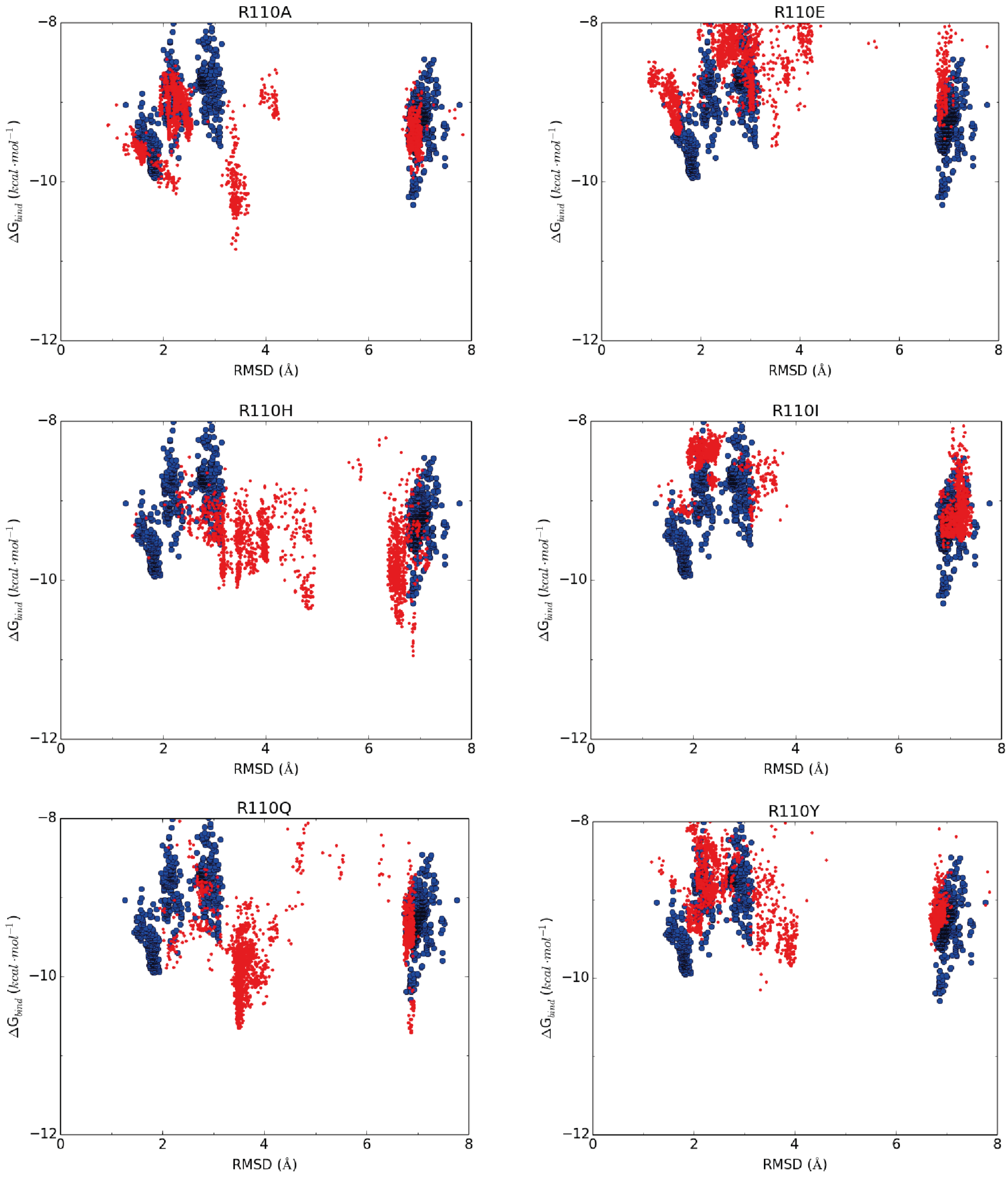

Figure S11. The RMSD-binding energy plots of mutants (red dots) at site 110 with respect to that of the wild-type (blue dots).

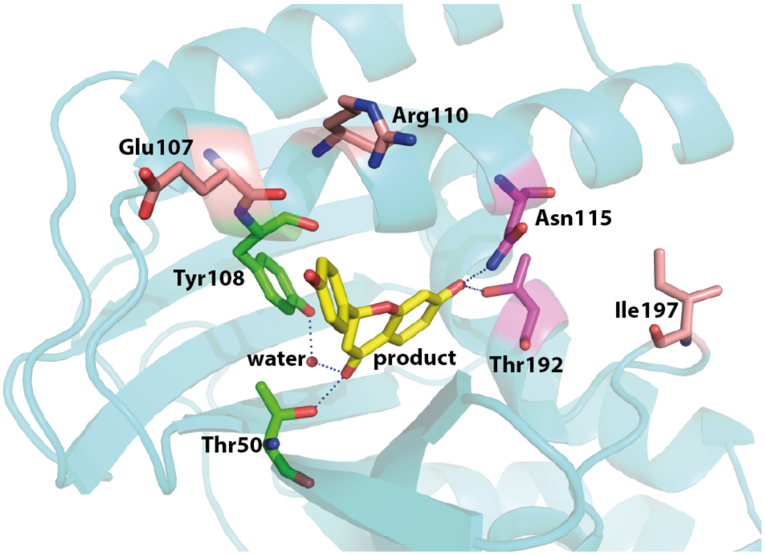

Figure S12. Spatial positions of the target mutation sites (Glu107, Arg110 and Ile197) at the active site.

S2.6. Enzyme assay

In this study, we used k_{cat} /Km to measure the catalytic proficiency. The standard Lineweaver-Burk equation is 1/v=(Km/Vm) × (1/[S]) + 1/Vm, and Vm = k_{cat} × [E]. To obtain k_{cat}/Km from the equation, we need to slightly transform the standard Lineweaver-Burk equation by multiplying [E] to both sides, and then we have 1/V = (Km/k_{cat}) × (1/[S]) + 1/k_{cat}, V = v/[E]. Now, the velocity (V) is defined as the generated moles of the product per second per mole enzyme during the catalytic reaction. So the k_{cat}/Km value could be obtained by taking reciprocal of the slope after linear fitting of 1/V ~ 1/[S].

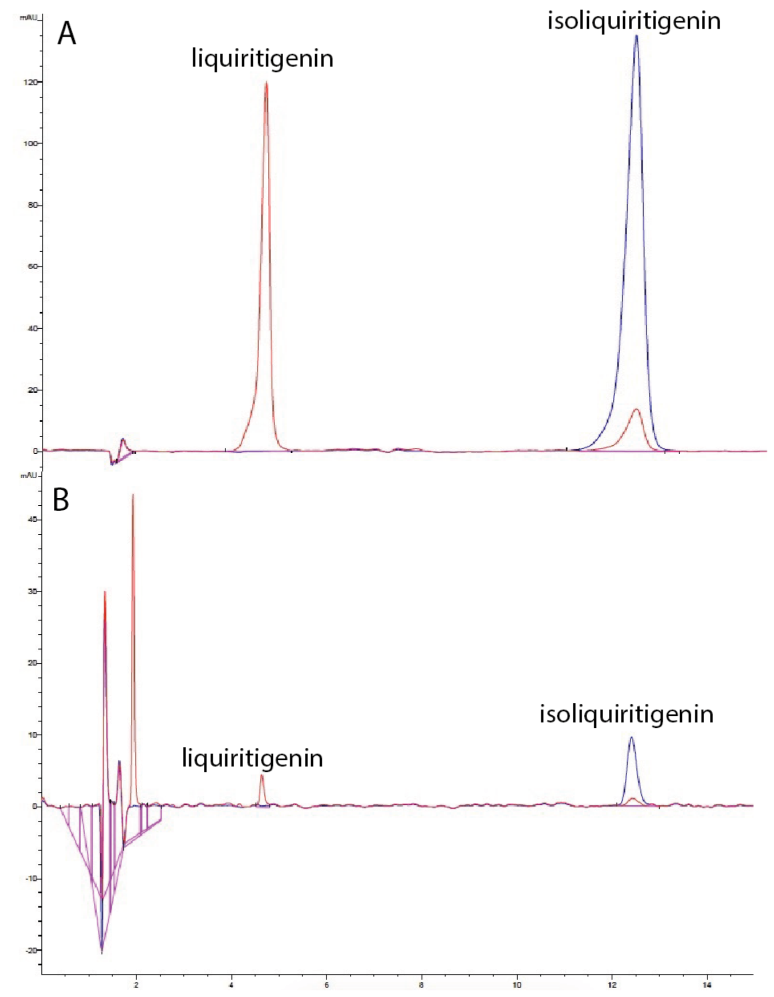

Figure S13. HPLC-UV analysis of isoliquiritigenin and liquiritigenin. (A) Reference standards. (B) Reaction mixture. The red line is for signal of 276 nm, and the blue line is for signal of 372 nm. The retention times for liquiritigenin and isoliquiritigenin are about 4.5 min and 12.5 min.

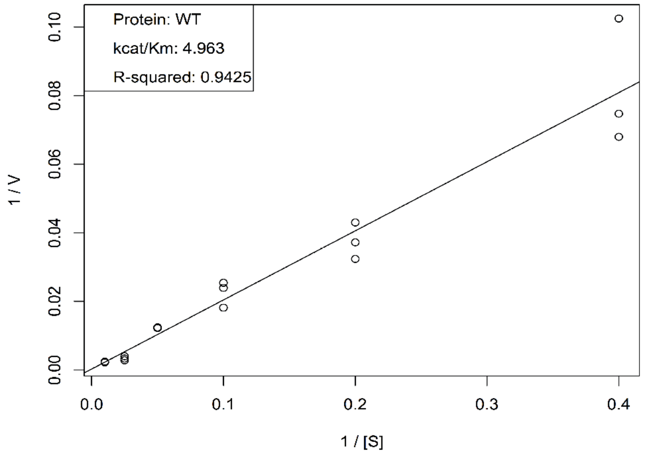

Figure S14. Lineweaver-Burk plot of the wild-type enzyme.

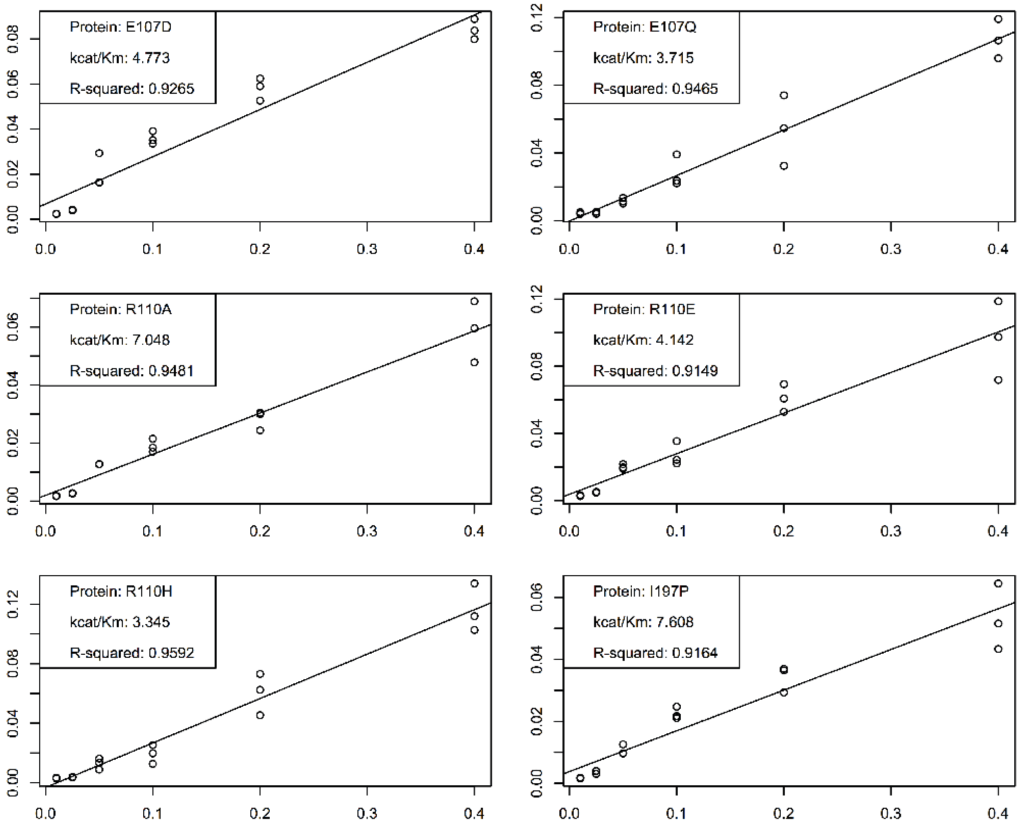

Figure S15. Lineweaver-Burk plots of six mutants (E107D, E107Q, R110A, R110E, R110H and I197P).

References

Eswar, N., Webb, B., Marti-Renom, M.A., Madhusudhan, M.S., Eramian, D., Shen, M.Y., et al. (2007). Comparative protein structure modeling using MODELLER. *Current Protocols in Protein Science* Chapter 2**,** Unit 2.9. doi: 10.1002/0471140864.ps0209s50.

Jez, J.M., Bowman, M.E., Dixon, R.A., and Noel, J.P. (2000). Structure and mechanism of the evolutionarily unique plant enzyme chalcone isomerase. *Nat. Struct. Biol.* 7(9)**,** 786-791.

Jez, J.M., Bowman, M.E., and Noel, J.P. (2002). Role of hydrogen bonds in the reaction mechanism of chalcone isomerase. *Biochemistry* 41(16)**,** 5168-5176. doi: Doi 10.1021/Bi0255266.

Jez, J.M., and Noel, J.P. (2002). Reaction mechanism of chalcone isomerase. pH dependence, diffusion control, and product binding differences. *J Biol Chem* 277(2)**,** 1361-1369. doi: 10.1074/jbc.M109224200.

Liu, C.J., Blount, J.W., Steele, C.L., and Dixon, R.A. (2002). Bottlenecks for metabolic engineering of isoflavone glycoconjugates in Arabidopsis. *Proc. Natl. Acad. Sci. U. S. A.* 99(22)**,** 14578-14583. doi: 10.1073/pnas.212522099.

Liu, C.J., and Dixon, R.A. (2001). Elicitor-induced association of isoflavone O-methyltransferase with endomembranes prevents the formation and 7-O-methylation of daidzein during isoflavonoid phytoalexin biosynthesis. *Plant Cell* 13(12)**,** 2643-2658.

Liu, C.J., Huhman, D., Sumner, L.W., and Dixon, R.A. (2003). Regiospecific hydroxylation of isoflavones by cytochrome p450 81E enzymes from Medicago truncatula. *Plant J* 36(4)**,** 471-484.

Shimada, N., Aoki, T., Sato, S., Nakamura, Y., Tabata, S., and Ayabe, S. (2003). A cluster of genes encodes the two types of chalcone isomerase involved in the biosynthesis of general flavonoids and legume-specific 5-deoxy(iso)flavonoids in Lotus japonicus. *Plant Physiol.* 131(3)**,** 941-951. doi: 10.1104/pp.004820.

Yang, Z.H. (2006). Neutral and adaptive protein evolution in *Computational Molecular Evolution*. Oxford University Press), 259-292.

Yang, Z.H., and Nielsen, R. (2002). Codon-substitution models for detecting molecular adaptation at individual sites along specific lineages. *Mol. Biol. Evol.* 19(6)**,** 908-917.

Yang, Z.H., Wong, W.S.W., and Nielsen, R. (2005). Bayes empirical Bayes inference of amino acid sites under positive selection. *Mol. Biol. Evol.* 22(4)**,** 1107-1118. doi: 10.1093/Molbev/Msi097.
